# ‘You’re not just a medical professional’: Exploring paramedic experiences of overdose response within Vancouver’s downtown eastside

**DOI:** 10.1371/journal.pone.0239559

**Published:** 2020-09-28

**Authors:** Jordan Williams-Yuen, Gordon Minaker, Jane Buxton, Anne Gadermann, Anita Palepu

**Affiliations:** 1 Department of Medicine, The University of British Columbia, Vancouver, British Columbia, Canada; 2 School of Population and Public Health, Faculty of Medicine, The University of British Columbia, Vancouver, Canada; 3 BC Centre for Disease Control, Vancouver, British Columbia, Canada; 4 Centre for Health Evaluation and Outcome Sciences, Faculty of Medicine, The University of British Columbia, Vancouver, Canada; Newcastle University, UNITED KINGDOM

## Abstract

**Background:**

Overdose response has become an increasingly relevant component of paramedic practice, particularly in light of increased opioid overdose globally. Previous studies have noted gaps in our understanding regarding the unique challenges which paramedics face during this form of pre-hospital emergency care. The aim of this study is to explore and describe the ways in which paramedics experience overdose response, specifically within a community markedly affected by the overdose crisis.

**Methods:**

Ten participants were recruited from a single ambulance station located in an urban center in Western Canada. Two rounds of semi-structured individual interviews were conducted, and data saturation was found to have been reached. Verbatim transcripts were produced and subject to two rounds of descriptive and pattern coding. A second researcher reviewed all of the codes, with disagreements being handled by discussion until agreement was obtained. Themes were identified, along with a Core Category which seeks to describe the underlying dynamics of overdose response represented in our data. The concept of a Core Category was borrowed from Grounded Theory methodology.

**Findings:**

Five major themes were identified: *Connecting with patients’ lived experiences; Occupying roles as clinicians and patient advocates; Navigating on-scene hazards; Difficulties with transitions of care; and Emotional burden of the overdose crisis*. A core category was identified as *One’s capacity to help*.

**Conclusions:**

This research contributes to existing literature on overdose response by specifically examining paramedic experiences during this form of emergency care. While paramedics felt highly confident in providing clinical care, their capacity to address underlying causes of drug use was understood as much more limited. Participants found ways to address this lack of control, along with feelings of frustration, by trying to understand patient perspectives and adopting empathetic attitudes.

## Introduction

Opioid use has emerged as a significant public health concern for countries worldwide [1–5]. The negative impacts of opioid dependency, most notably overdose, has led to a global response involving calls to action from both the United Nations and World Health Organization [6,7]. North America has experienced the bulk of global overdose deaths, with mortality rates being high enough to lower overall life expectancies in both Canada and the United States [8,9]. The province of British Columbia (BC) has been an epicenter for Canada’s struggle with the overdose ‘crisis’, and declared the situation to be a public health emergency in 2016 [10–12]. BC accounts for approximately one third of national overdose deaths, despite representing only 13% of the population [13,14].

As front-line healthcare workers, paramedics are directly impacted by the overdose crisis. Qualitative studies have noted that heightened incidence of opioid overdose has led to feelings of increased professional demand amongst first responders [15]. The workload associated with overdose response can be significant; BC paramedics attended 13,500 overdose events in 2019, a value which has more than doubled since 2015 [13]. Overdose response, as well as the associated overdose crisis, has been linked to burnout amongst paramedics and Emergency Medical Service (EMS) providers [15,16]. This emotional strain is occurring on an already at-risk population, as paramedics exhibit markedly higher rates of post-traumatic stress disorder (PTSD) than the general population [17].

Qualitative exploration builds upon the context of experiences outlined by clinical guidelines and protocols. Governing organizations for paramedics emphasize airway management, oxygenation, ventilation, and transport as key principles of overdose response [18, 19]. Although many North American health authorities have allowed firefighters or police to administer naloxone when encountering overdose [20–23], the medical training and protocols available to paramedics exceeds those typically available to other first responders [24, 25]. Thus, paramedics are uniquely situated amongst other pre-hospital emergency personnel due to their expanded medical scope of practice, as well as their role in transporting patients to further medical care.

Previous studies have examined healthcare worker experiences when treating overdose, however there has yet to be a focused exploration of paramedic perspectives on this topic [26]. As the primary providers of pre-hospital medical care, paramedics are uniquely situated amongst other emergency personnel. Differences have been noted between paramedics, fire fighters, and law enforcement officers regarding attitudes towards overdose response, and this has led to a call for occupation-specific investigations [15,26]. Understanding the unique challenges and concerns of paramedics can inform the types of support provided to these frontline workers amidst an ongoing public health emergency. The goal of this study is hence to explore the experience of paramedics during overdose response, specifically within a community markedly affected by the overdose crisis.

## Methods

### Study design and recruitment

We conducted semi-structured qualitative interviews with 10 paramedics in Vancouver, British Columbia. Participants were recruited between May and June 2019 from a single ambulance station within the city’s downtown eastside. This region of Vancouver experiences the highest levels of overdose events and deaths within the city, as well as the province at large [13, 27].

An email introducing the study and inviting volunteer participation was sent to all full-time paramedics at the station. Posters with the same information were also set up in common areas around the station. Paramedics who responded were provided with additional information about the interviews, including the expected time commitment, format, and confidentiality protocols. A detailed consent form was reviewed in-person with each participant, and signed, prior to beginning the interview.

Interviews were conducted with the first six paramedics who responded to the email or poster invitations. Demographic information from these participants was reviewed; we identified under-representation of paramedics with higher seniority and older age. The unit chief was consulted to identify station paramedics who could potentially fill these gaps. These paramedics were contacted via email to ask about their interest in participation, and 4 additional interviews were arranged.

### Ethics

This study received ethics approval from the UBC-Providence Healthcare Research Ethics Board (approval number H19-00853). It also received research partnership approval from the BC Emergency Health Services (BCEHS) Pharmacy, Therapeutics, Research and Practice Advisory Council (PTRPAC).

### Interviews and analysis

The lead author (JWY) worked as a part-time paramedic for two years prior to beginning this study, and was also studying as a medical student during data collection and analysis. As a result, JWY occupied both familiar and unfamiliar roles relative to the more-senior paramedic participants. Measures were taken to address reflexivity and possible bias related to these positions [28]. These included: engaging in discussions and self-reflections which examined JWY’s own expectations and experiences related to the study [29], conducting repeat interviews with participants [30], and involving a multi-disciplinary team which included non-paramedics [31]. All participants were informed of JWY’s paramedic background prior to conducting interviews.

An interview guide was developed prior to conducting the interviews (S1 File). This guide was informed by the researcher’s experience working as a paramedic as well as existing literature on emergency responder experiences [32, 33]. One pilot interview was conducted by JWY with a part-time paramedic in order to assess whether questions were phrased clearly for the participant, and also to allow the researcher to become familiar with using the interview guide in practice [34, 35]. This pilot interview was not included in the final analysis as the participant had limited experience working from the station included in this study.

Interviews were conducted individually in private settings by JWY, most commonly offices in ambulance stations, and typically took between 45 to 60 minutes. Participants were put into a raffle for one of two restaurant gift certificates. No other honorarium was provided to the participants.

Audio recordings were transcribed verbatim by the lead author. Descriptive coding was performed on each new transcript in order to identify broad topics which were being discussed in interviews [36]. After 10 participants were interviewed, it was observed that the same topics were being repeatedly brought up across multiple participants. Data saturation was determined to have been reached based on this redundancy of subject matter [37]. The NVIVO computer program, version 12.5, was used to organize data and facilitate coding.

Follow up interviews were conducted to deepen the data on topics which were identified as central through a first round of descriptive coding, and also to ensure complete coverage of those topics amongst participants. For example, the topic of ‘patients who refuse transport’ was identified as a common experience despite not explicitly being part of the interview guide. Follow up interviews would invite participants to discuss their experiences on this topic if they did not do so during the first interview. Read [38] describes the process of repeat interviews as a way to add depth and breadth to qualitative data, particularly when the research question involves ‘multiple or multidimensional topics.’ Individual follow-up telephone interviews with all participants were conducted and lasted approximately 20 minutes. Content from the follow-up interviews was similarly subject to an initial round of descriptive coding.

Further analysis was informed by the approaches described by Saldana [36]. Using the descriptive codes as a baseline, a second cycle of pattern codes were produced which described more nuanced qualities of the data. At the same time, an analytic memo was developed which highlighted notable quotes and attempted to summarize emerging trends in the data [39]. The purpose of this document was to help the researcher critically reflect on the findings while remaining grounded in content from the transcripts.

A second researcher (GM) with previous qualitative research experience was introduced after the second cycle of codes were developed. GM was working as a part-time paramedic during this period in addition to being a third-year medical student. GM reviewed all the transcripts independently before discussing the appropriateness of each code with JWY. Disagreements were handled by discussion until consensus was achieved. Changes to the codes were made based on these discussions and new insights. The next stage of analysis involved rearranging the codes into six new clusters that reflected higher-order concepts. These represent the themes presented in this paper. Finally, a core category was identified which sums up 'the substance of what is going on in the data' [40]. A model was developed around the core category, and this was also shared to GM for further discussions and adjustments. The concept of a core category was borrowed from grounded theory methodology, which aims to produce descriptive models centered around a fundamental theme or category [40].

### Findings

Ten paramedics were interviewed before data saturation was reached (Table 1). There were 5 male and 5 female participants, with a mean age of 49 years. Nine participants were full-time employees, while one participant was a part-time employee working out of the Vancouver/North Shore district. This employee had significant experience working in the relevant region, and hence was considered an appropriate participant to include in the study.

**Table 1:** Demographics of study paramedics.

	Males	Females	Total
	N = 5	N = 5	N = 10
Mean Age (years)	52	46	49
Mean time working as a paramedic (years)	23	14	18

Five major themes were identified; *Connecting with patients’ lived experiences; Occupying roles as clinicians and patient advocates; Navigating on-scene hazards; Difficulties with transitions of care;* and *Emotional burdens of the overdose crisis*. The core category of *Control over one’s capacity to help* was identified to describe the underlying dynamics of overdose response represented in our data. There were no identified differences between genders in terms of trends in responses.

### Theme 1 –Connecting with patients’ lived experiences

Participants in this study recognized addiction to be a complex mixture of biological, psychological, and social factors. Listening to patients' lived experiences was described as an important aspect of overdose response, and a process which helped them to understand patients’ perspectives of drug use and addiction.

After a patient had been medically resuscitated from an overdose, participants had the opportunity to engage with them personally. Participants described instances where they were able to connect with overdose patients as they transported them to hospitals or local clinics: “*you sit in the back of the ambulance and you can*, *not always*, *but talk on the way*. *Everybody has a story*, *right*?” (Paramedic B2) These experiences were described as formative to participants’ views on drug use and addiction.

*“A good majority of that attitude I have about it has developed strictly from just talking to patients…I’ve asked patients ‘what is it like being dope sick?’ Talk to me*. *Really talk to me about it…What’s your story?” (Paramedic A1)*

Listening to the 'stories' of overdose patients was a commonly cited aspect of the post-resuscitation experience. The process of continually being exposed to those stories was described as one way to appreciate the diverse circumstances which can lead to addiction.

"*I think I've learnt from just talking to people that drug addictions happen to people for all sorts of different reasons*. *From all sorts of different backgrounds*, *areas*, *genders*. *And just the trauma that brings on*, *that drives addiction*, *is heartbreaking*.*"* (Paramedic A2)

The idea of experiential learning, or “*seeing it with my eyes*” (Paramedic E1), emerged as a common phrase among participants. Working within the Downtown Eastside neighborhood and responding to overdose calls was described as “*eye opening*” (Paramedic C2) experiences that “*opened my eyes to what the people’s perspective is*.” (Paramedic H1).

Participants often described perspectives of their work through encounters they had with people who were not experienced with overdose response. These included paramedic students early in their career, or family members who are not paramedics:

*“I’ve heard some people say that we shouldn’t have Narcan [i*.*e*. *naloxone]…that by giving people Narcan we’ve taken away that risk and now there’s no end to the problem…So I hate to say*, *it but I think I may have had that feeling when all of this first started and I didn’t have much experience with it*.*”* (Paramedic A2)

### Theme 2 –Occupying roles as clinicians and patient advocates

Participants identified their roles during overdose response as being both clinician and patient advocate. These roles are informed by the basic pillars of paramedicine: medical care and transportation. However, participants placed additional meaning onto these roles and described how they play out in the context of overdose response.

Participants identified clinical assessment and resuscitation as fundamental aspects of their role in overdose response:

*“We're there for [patient] safety*. *They're not breathing*, *we correct that*. *We go and treat them*, *and make them safe again*.*"* (Paramedic H1)

Participants saw themselves as the clinical specialists in a pre-hospital setting: “*I have the skills and I have the training”* (Paramedic E1). They expressed a high degree of confidence towards their ability to assess and treat overdose patients. Many participants experienced overdose response as a relatively simple call to run:

*"It’s just ABCs you know [airway*, *breathing*, *circulation]*. *It’s one of the calls where I find sort of the easiest to run because here’s the problem*, *we’re working on it*, *we’re working on it*, *and they get up*. *Most of the time it follows this very regimented script*.*"* (Paramedic A2)

The emotional experience of overdose response was partly tied to this clinical role and their associated responsibilities. Participants described negative experiences when they were unable to fulfill the responsibilities placed on themselves as clinicians:

*“A success would be if you did your best with the equipment and the medications you have…what feels negative to me*, *if the call didn’t go well*, *is if I missed something*. *If I didn’t do something*.*”* (Paramedic H1)

Participants noted that it was very common for paramedics to arrive at overdoses where bystanders are already involved in clinical procedures such as naloxone administration or ventilation. In these cases, participants experienced their clinical role through a leadership lens. Participants described “*coaching*” (Paramedic F1) members of the public in proper ventilation technique and developing a “*cooperative approach*” (Paramedic G1) where the paramedic would “*double check*” (Paramedic A2) the effectiveness of bystander involvement.

Patient advocacy was identified as a second important role of paramedics during overdose response, and one which was separate from clinical assessment or protocols: “*You’re not just a medical professional*.*”* (Paramedic A1) The term ‘advocacy’ was used in a variety of contexts to describe paramedics using their position as healthcare workers to access resources for patients. Participants experienced this role once patients were alert and medically stable: “*because that’s sort of phase two of the call*.” (Paramedic D1)

Participants described trying to gain access to a wide variety of resources for their patients. The most commonly cited medical resource was treatment programs for addictions, however, participants also associated advocacy with trying to obtain basic needs such as blankets, meals, or places to stay for a night. One participant succinctly summarized their role as a patient advocate: “*my role is to try and get that guy some help*.” (Paramedic A1)

Sometimes advocacy involved making decisions which prioritized patient needs above regular protocols. One participant described an experience where they decided to transport a patient who had overdosed to a second hospital. This decision was made after another healthcare worker complained that the patient had already been to the hospital a day earlier.

*“…and I think ‘oh man*, *I can’t just leave this guy here” …My role isn’t necessarily to say ‘hey listen*, *you’re drug seeking*.*’ That’s not my job*. *My job is to advocate*, *whatever that may look like*. *And sometimes it may look weird*.*”* (Paramedic B2)

### Theme 3 –Navigating on-scene hazards

Encountering and adapting to on-scene hazards was described as a routine aspect of overdose response. Paramedics relied on their situational awareness, interpersonal skills, and occasional assertiveness to prevent harm to themselves or others.

Participants noted that during an overdose response a variety of safety risks may be encountered. They described two categories of hazards: immediate risks, such as uncapped needles or patients who are agitated due to withdrawal following resuscitation, and potential risks such as working in cramped spaces, presence of weapons, or bystanders. Immediate hazards were not always perceived as more worrying than potential ones, as a paramedic’s ability to control a given hazard factored into their sense of safety:

*“Needles all over the floor I can deal with because I know that they’re there* …*I can deal with that*. *I don’t like the guy sitting in the chair*, *while we’re doing an overdose in the SRO’s [single room occupancy hotels]*, *staring at me*, *with the knife next to him*. *That bothers me*.*"* (Paramedic A1)

Participants described behaviors they adopted to respond to the risks encountered during overdose response. Situational awareness was upheld as an important aspect of safety management, with participants citing the value of taking a “*big picture approach*” (Paramedic C1), or keeping “*360-degree coverage as much as possible*.” (Paramedic F1). Participants noted that overdose resuscitation requires paramedics to monitor small details about their patients. This can then make it challenging to maintain awareness of your surroundings:

“*But my head is on a swivel*, *right*. *I’m looking at the patient*, *looking at the numbers*, *always checking around*.*”* (Paramedic A1)

Participants also described the use of respectful, non-judgmental attitudes as a way to mitigate risks of violence from bystanders or patients: “*I show up and I treat people the way I wish I would be treated*, *which calms things down right there*.” (Paramedic D1) Some participants found it helpful to remind themselves of the physiological basis of post-overdose aggression, particularly when patients presented with naloxone-precipitated opioid withdrawal: *“Some people are going to be a little angrier*. *So you have to understand that it’s a medical problem*.*”* (Paramedic G1) One participant learnt to recognize the influence of their actions, which ultimately led to a sense of ownership over safety outcomes.

*“In almost all of the scenarios where…I have been challenged or confronted…on reflection*, *I realized it was probably something I said or did intentionally or unintentionally that triggered that response*. *And so you adjust and adapt*, *and carry on*.*”* (Paramedic F1)

When these de-escalating approaches were not sufficient, paramedics found it necessary to become “*assertive*” (Paramedic A1 and E1) or “*take charge*” (Paramedic A2) of the situation.

The emotional experience of safety was varied across participants. Some paramedics felt that there was a degree of unavoidable risk associated with overdose response: “*do I feel safe*? *No*. *But you can only ameliorate it so such a degree as you can*.” (Paramedic C1) One participant experienced a need to overcome, or “*push*” (Participant B2), through their feelings of hesitancy in order to respond to certain overdose calls.

In contrast, other participants expressed feeling quite safe during overdose calls, and that it was “*very rare*” (Paramedic D1) for them to feel otherwise. For one participant, even being presented with an immediate threat of violence was not enough for them to experience a lack of safety:

*“I had one a couple blocks ago where the guy came up swinging and decided to come after me*, *and the firefighters just closed ranks because he was literally coming after me*. *And we called for the cops*. *But I didn’t feel afraid*, *afraid*. *It was just uncomfortable*.*”* (Paramedic D1)

### Theme 4 –Difficulties with transition of care

Participants regularly experienced challenges in transitioning patients who had overdosed to other forms and levels of care. Participants had to negotiate patient and paramedic perspectives to decide where to transport their patients, if at all. Uncertainty remained as an important aspect of this experience, due to lack of patient follow- up after transition of care.

In order to transition care of their overdose patients, paramedics had to first determine a destination to transport to. Options included hospitals, overdose supervision clinics, or no transport at all. Participants consistently described hospitals as the ideal place to transfer care of overdose patients. This was primarily due to their ability to provide medical supervision, particularly to account for the possibility that the effects of Naloxone wear off and increase the risk of overdose recurrence. The ability to access addiction treatment was another important factor to the hospital’s appeal. However, participants recognized that there were “*some considerations to look at*.” (Paramedic B2)

These considerations centered around patient preferences and perspectives; participants frequently encountered overdose patients who would refuse transport or express hesitancy in going to the hospital. Precipitating opioid withdrawal further complicates the situation, as patients “*might not make the best rational decisions when they wake up*.” (Paramedic G1)

When discussing next steps of care with patients, participants had to navigate tensions between their own preferences and the “*myriad of different reasons*” (Paramedic A2) which made overdose patients hesitant to visit the hospital. Participants recognized that stigma against overdose patients can lead to providers "*judging them*" (Paramedic F1) within healthcare spaces. Participants also noted that hospitals primarily provide medical supervision to overdose patients, as opposed to direct treatment, which can feel less valuable from a patient perspective.

Discussing care options with patients was often experienced as a type of negotiation process. Participants described trying to “*entice*” (Paramedic C1) overdose patients by describing some benefits of hospital care: blankets, food, access to social workers. Some participants described being heavily invested in these discussions: “*sometimes it just feels like I am begging*.” (Paramedic D1) For some participants, offering transport to Overdose Prevention Sites, where supervision is offered in a non-hospital setting, was a form of compromise if hospital transport was unrealistic: “*If they are refusing transport*, *that’s when I try to go with one of the other places we can transport*.*”* (Paramedic A2)

Ultimately, participants recognized that paramedics must respect the right of overdose patients to refuse transport if they are competent to do so. While some participants described being at peace with this situation, frustration and other negative emotions were commonly cited. One paramedic described how their emotional experience changed as they became accustomed to the occurrence;

*“At first it was heartbreaking…the first twenty*, *thirty overdoses you go to that are code X’d [refused transport] by the patient*, *you feel a little disheartened afterwards*. *But now*, *it becomes such a regular occurrence that there is not too much emotional attachment to it*.*”* (Paramedic A1)

The transition of patient care from paramedics to hospital workers was often characterized by uncertainty, speculations, and lack of closure. Participants noted that they have limited capacity to follow-up with their patients, and so are unlikely to see long-term outcomes of overdose patients who are interested in treatment programs for addiction;

*“It’s not often you get to have this…this wonderful response that gives me an idea that something is going to be positive*. *Because you’re not around the patient once they get to the hospital*. *And who knows what happens to them afterwards*.*”* (Paramedic C1)

Similarly, one participant described how enrollment into addiction treatment programs is dependent on down-stream actions of healthcare workers who take over care of their patient. As a result, there is always uncertainty as to whether paramedic advocacy efforts are successful:

*“It’s like*, *here are the resources in this building*. *I’m going to advocate the hell out of this*, *and I hope to god that the triage nurse*, *or the bedside nurse*, *or the bedside doctor gets what I am trying to say*.*”* (Paramedic D1)

### Theme 5 –Emotional burden of the overdose crisis

Participants described the emotional stress they have experienced from changes associated with the overdose crisis. Encountering the same patient repeatedly was a common occurrence and associated with a wide range of challenging emotions. The increased volume of calls which paramedics respond to was also cited as a source of stress which interfered with their capacity to emotionally process stressful encounters.

Interacting with repeat overdose patients was described as an emotionally-charged aspect of overdose response, and one which had become normalized by the overdose crisis. Participants described feeling frustration and resentment during these encounters, with one paramedic labeling their experience as “*compassion fatigue*” (Paramedic C1). This was in part brought about from the feeling that the care they were providing was insufficient: “*we’re just going out and we’re dealing with a symptom*. *We’re not dealing with the issue*.*”* (Paramedic C1)

When discussing patients who repeatedly overdose, feelings such as compassion or sadness were oftentimes co-expressed alongside a recognition of frustration. These emotions were grounded in an understanding that repeat overdose patients are oftentimes struggling with complex needs that are both medical and psychological:

*“It can be frustrating*, *but at the same time*, *there are so many other things that aren’t taking place…It’s this endless cycle*. *It’s sad to me*.*”* (Paramedic C2)

Participants struggled with the mental toll of being exposed to overdose deaths brought about by the crisis. One paramedic described how that mindset had carried over into their encounters with repeat patients:

*“You kind of go*, *‘this guy who I see on a regular basis*, *I need to prepare myself that sometime in the next six months I’m not going to see him anymore*.*’ And not think about it*.*”* (Paramedic D1)

Participants also described how increased volume of overdose calls had been its own source of stress. One participant painted a vivid picture of the chaos associated with these call volumes:

*“I remember when this whole crisis first started…there was probably about a four-block radius…where I counted 8 ambulances doing overdoses…And I stopped*, *and I looked around*. *Ambulance there*, *ambulance there…And you could hear sirens coming in*, *and it was just like ‘what is going on*?*’…It was completely overwhelming*.*”* (Paramedic A1)

Rates of overdose calls were felt to be concentrated around social service payments, nicknamed ‘welfare Wednesdays’:

*“it’s just overdose*, *overdose*, *overdose…It’s hard and fast and relentless*.*”* (Paramedic D1)

The pace of these shifts was described as challenging in part because they allowed no “*time to process*” (Paramedic D1) some of the more emotionally challenging calls:

*“We do some many calls…I need to go out to the next one*, *and if I carry too much it builds up*, *especially after the fourth shift*.*”* (Paramedic B2)

### Core category—one’s capacity to help

The concept of a core category is taken from grounded theory methodology, and represents a category which sums up “the substance of what is going on in the data” [40]. Specific characteristics of core categories include: relating to a number of other categories or themes, relating easily to those themes, and reoccurring frequently throughout the raw data [40].

The core category identified in our data was *One’s Capacity to Help*. This category refers to the extent in which paramedics feel capable, or incapable, of addressing the needs of patients who overdose. Participants related many of their experiences back to this central concept, which incorporates ideas from many of the previously-described themes.

Connecting with patient stories was described as an important way in which participants gained perspectives on the psychosocial causes of addiction. However, although participants were able to recognize the complex needs of patients who overdose, they did not always feel capable of addressing those needs;

*"I think I’ve always known that I can reverse an overdose*, *but I can’t make someone not be poor and do drugs*.*”* (Paramedic C2)

The distinction between providing medical and non-medical care was reflected in how participants experienced their roles during overdose response. Safety hazards were described as significant in part because they interfered with the ability of paramedics to fulfill their clinical roles. Participants described tensions or conflict when their capacity to help was limited by on-scene hazards:

*“You want to jump in there and you want to save the person*, *but you don’t want to do that in the peril of your own safety*.*"* (Paramedic B2)

While participants were ultimately very confident in their ability to provide clinical support, "*it's like second nature*" (Paramedic E1), the capacity of paramedics to provide non-clinical forms of support was described as much more tenuous. Participants noted that their ability to access social services or addictions treatment programs was reliant on the willingness of patients to be transported after overdose. However, this dynamic often felt outside of their control:

*"I try to do my best to get [overdose patients] the help they need*, *but if somebody has it made up in their head that they are not going to go to the hospital*, *then I don’t think there is much I can do to change their mind* …*When I first started*, *it really bothered me*.*"* (Paramedic A2)

The emotional experiences of paramedics were tied to their capacity to help patients. Participants described positive experiences when patients gave them the opportunity to engage in patient advocacy:

*“It’s a super victory* …*when they go ‘you know what…I think I need to go to detox*.*’ And it’s like ‘Okay*, *let me hook you up*.*’…And I cheer them on*.*”* (D1)

Relatedly, experiences which affirmed the impacts of patient advocacy work were described as emotionally powerful:

*“I had him for overdoses for maybe six of them…And I said ‘listen man* …*this isn’t working for you anymore*. *Let’s get you off the heroin*.*’ And he came with me*, *and I ran into him six month later… He said to me*, *‘I have not touched the heroin since the day you brought me in*.*’ And I felt like crying*.*”* (Paramedic A1)

Encountering repeat patients who overdose was described as an experience which often left participants feeling helpless. However, while paramedics frequently felt unable to provide care that would end the repeating cycles they witness, their experiences were not defined entirely by this sense of disempowerment. Some participants described ways in which they found meaning, or a sense of accomplishment, in situations where they might otherwise feel no control over. For example, one participant described how focusing on emotional support helps them feel like they have provided meaningful care:

*"I offer kindness*. *And often I just listen* …*It sounds kind of weird*, *but offer a glimmer of hope* …*I think it helps the patient by helping me…So then I don’t feel so bad*, *I feel like I’ve done something*. *And it puts us on equal footing…You are not a ‘drug addict’ and I’m not here ‘saving you’*.*"* (Paramedic B2)

Similarly, one participant framed the experience of providing care to repeat patients as an opportunity to build connection. This would ultimately help the paramedic facilitate hospital transport in the long-term:

*"Knowing their name*, *or even them knowing you* …*is such a huge leap forward in terms of being able to talk that person into going to the hospital*. *If they feel that you care about them*, *or have that rapport with them*, *they are going to be way more open to listening to what you have to say*.*"* (Paramedic A2)

One participant summarized how they worked through feelings of helplessness encountered while treating repeat patients during overdose response. Their approach frames paramedic frustration and stigma as reactions to their own emotional vulnerability. For this participant, connecting deeply with patient experience allows them to provide compassionate care in situations which might feel hopeless:

*“You are trying not to*, *but you have built a wall because you want to defend yourself from feeling too much from somebody who can’t help shooting up again*. *So understanding what substance use disorder is*, *like really understanding and seeing it first-hand*, *you can still empathize but you don’t actually have to take it on…But it takes time*. *It’s not something that you can just switch*, *and change your ideology*. *But if you get past that–that’s where you actually are able to have compassion all the time*. *Without feeling overwhelmed*.*”* (Paramedic G1)

## Discussion

The purpose of this study was to explore the ways in which paramedics working within an urban center experience overdose response. Whereas similar qualitative studies to date have included paramedics within a larger pool of emergency personnel [15, 26], this study brings a singular focus onto the perspectives of paramedics specifically. Furthermore, this study details experiences from a region with markedly high overdose rates relative to both Canadian and North American averages [2,13,27].

Pike (2019) found that, compared to law enforcement officers, EMS providers were more likely to believe the overdose crisis has contributed to burnout in their field. One suggested explanation from the authors was that the advanced medical involvement of paramedics could lead them to experience more traumatic events during overdose response. However, participants in our study did not identify paramedic clinical roles as significant causes of emotional stress. Instead, distress was associated with negative interpersonal interactions, as well as broader feelings of helplessness. While participants in our study discussed dynamics, which may appear to be paramedic-specific, such as the process of transporting patients, this study did not directly compare experiences between paramedics and law enforcement officers. Studies which compare these two fields more directly may be necessary to explain differences in burnout.

Previous qualitative studies of emergency personnel experience have largely reported on negative aspects of overdose response. Indeed, participants in our study identified similar challenges, notably encountering repeat patients and patients who refuse transport [15,26]. Similarly, the core category we identified reflects previous findings of helplessness as a central, negative emotion during overdose response. However, participants in our study also outlined positive experiences, for example encounters with patients who are open to receiving treatment for addiction, and follow-up encounters with those patients. Our study was also able to identify compassionate attitudes towards repeat patients, and approaches which found meaning in these potentially-frustrating circumstances. These findings contribute to a deeper understanding of overdose response within the literature. They document novel narratives of paramedics finding purpose and empathy within an experience that has largely been represented as triggering hopelessness.

Studies involving healthcare workers have described high levels of empathy as a protective factor against burnout among healthcare workers [41–43]. However, researchers have noted a lack of studies investigating this dynamic within paramedic populations [44]. While our study does not directly investigate burnout, participants did describe empathetic attitudes as a form of resilience against feelings of helplessness. Given that helplessness, and a general lack of control, has itself been associated with burnout [45], our findings suggest that further study into these relationships may be valuable. Future research should explore paramedic resilience during the overdose crisis with a particular emphasis on how empathy and experiential learning contribute to that process.

## Strengths and limitations

Paramedics in this study were recruited from a single ambulance station situated in a unique location of British Columbia. Vancouver's downtown eastside is known to experience high rates of overdose events, poverty, mental illness, housing instability, and substance use [46–48]. The site of this study was purposefully chosen because paramedics working there are likely to have a great deal of exposure to overdose calls. However, a consequence is that the expressed experiences may be less generalizable to other (e.g. rural) settings.

The participant pool for this study was relatively small, however, it did demonstrate equal gender representation and a wide range of seniority levels. Sampling continued until there was redundancy of ideas within interviews, suggesting that saturation was still seen to be reached despite the modest number of participants.

Both GM and JWY have experience working as paramedics, and therefore the impact of that positioning should be considered. Shared experiences with participants can help facilitate honest discussion during interviews, however it can also act as a biasing lens which information is filtered and interpreted through [28]. Researchers attempted to mitigate this bias by discussing findings with a multi-disciplinary team, which included co-researchers that were not paramedics or did not come from a medical background. By interrogating the findings from multiple perspectives, researchers attempted to minimize the risk of imposing a single bias, belief, or attitude onto the data [29].

## Conclusion

This research contributes to existing literature on overdose response, and does so by specifically examining paramedic experiences during this form of emergency care. Participants identified their roles as both clinicians and patient advocates. While paramedics felt highly capable of providing clinical care, their capacity to address underlying causes of drug use was understood as much more limited. Participants found ways to address this lack of control and feelings of frustration by trying to understand patient perspectives and adopting empathetic attitudes. Future research should continue to explore the unique ways in which paramedics have been impacted by the overdose crisis, with a particular emphasis on how empathy and compassion might relate to paramedic resilience.

## Supporting information

S1 File Interview guide. Interviews were semi-structured and used this guide as a basis for questions.
(DOCX)
