# Supplementary material for: ‘You’re not just a medical professional’: Exploring paramedic experiences of overdose response within Vancouver’s downtown eastside
Source: PLoS One. 2020 Sep 28;15(9):e0239559. doi: 10.1371/journal.pone.0239559 (PMC7521748; doi:10.1371/journal.pone.0239559)
Supplement: S1 File — Interviews were semi-structured and used this guide as a basis for questions. (DOCX) [file pone.0239559.s001.docx]

**Interview Guide Questions**

**Exploring Experiences During Overdose Response**

*Cognitive*

- What do you think is your role as a paramedic when responding to an overdose?
- What are your priorities when responding to an overdose?
- What are some risks during an overdose emergency response? How do you manage risk?
- To what degree can you collaborate with bystanders or civilians during an overdose response?
- Is there anything that could be done to improve your experience during overdose response?

*Affective*

- What sorts of emotions have you felt during an overdose response?
- What would happen for an overdose response call to feel like a ‘success’?
- Do certain types of overdose response calls feel less rewarding than others?
- What are the most challenging aspects of overdose response?
- In what sorts of environments do you feel most comfortable responding to an overdose? What environments might make you feel the least comfortable?

**Exploring Attitudes Towards Drug Use**

*Cognitive*

- In your opinion, what are some reasons why an individual would begin or continue to use illicit drugs?
- To what degree do you think continuing to use drugs is within someone’s control?
- What is your role, as a paramedic, in supporting people who use drugs?

*Affective*

- When you consider providing care to people who use drugs what sort of emotions come to mind?
- Have there been any experiences you have had with people who use drugs that have provoked a strong emotional response?

**Exploring Connections Between Experiences and Attitudes**

- What sorts of experiences have shaped your view of drug use? Do you think your views about drug use have changed over time?
- Do you feel that your attitude towards providing care for people who use drugs has changed over time? (Probe - if yes, how and if no, why)
- How do you feel that your attitudes toward drug use affect your approach to overdose response?
